# Influence of Temperature on Growth and Production of Pectenotoxin-2 by a Monoclonal Culture of *Dinophysis caudata*

**DOI:** 10.3390/md13127061

**Published:** 2015-12-03

**Authors:** Leila Basti, Hajime Uchida, Ryoji Matsushima, Ryuichi Watanabe, Toshiyuki Suzuki, Toshifumi Yamatogi, Satoshi Nagai

**Affiliations:** 1 Department of Ocean Sciences, Tokyo University of Marine Science and Technology, Minato, Tokyo 108-8477, Japan; 2 National research Institute of Fisheries Science, Fisheries Research Agency, Fukuura, Kanazawa, Yokohama, Kanagawa 236-8648, Japan; tsuzuki@affrc.go.jp (H.U.); matsur@affrc.go.jp (R.M.); rwatanabe@affrc.go.jp (R.W.); tsuzuki@affrc.go.jp (T.S.); 3 Nagasaki Prefectural Institute of Fisheries, 1551-4 Taira, Nagasaki, Nagasaki 851-2213, Japan; yamatogi011143@pref.nagasaki.lg.jp (T.Y.)

**Keywords:** *Dinophysis caudata*, culture, growth, pectenotoxin-2, temperature

## Abstract

The effects of temperature on growth and production of Lipophilic Toxins (LT) by a monoclonal culture of *Dinophysis caudata* was studied. The cell density of *D. caudata* increased significantly with increasing temperature, and was the highest under 27, 30, and 32.5 °C. Temperature affected the average specific growth rate (µ) during the exponential growth phase (EG), which increased from 15 °C to 30 °C, and then decreased at 32.5 °C. Liquid chromatography-tandem mass spectrometry (LC-MS/MS) revealed that this strain of *D. caudata* produced only pectenotoxin-2 (PTX-2) whose concentration increased significantly with incubation period, except at 32.5 °C. It was significantly different between temperatures ≤18 °C, ≥21 °C, and 32.5 °C. The cellular toxin production (CTP, pg·cell^−1^·day^−1^) showed variation with growth phase and temperature, except at 32.5 °C. The average net toxin production (R_tox_) was not affected by temperature. During EG, the average specific toxin production rate (µ_*tox*_) increased significantly with increase in temperature, reaching a peak of 0.66 ± 0.01 day^−1^ at 30 °C, and then decreased. Over the entire growth span, µ_*tox*_ was significantly correlated to µ, and this correlation was most significant at 27 and 30 °C. During EG, µ_*tox*_ was affected by both temperature and growth. This study shows that temperature affects growth and toxin production of this strain of *D. caudata* during EG. In addition, a positive correlation was found between toxin production and growth.

## 1. Introduction

The human seafood-borne intoxication known as diarrheic shellfish poisoning (DSP) was first identified in Japan in the 1970s [1]. It is associated with the consumption of bivalve molluscs contaminated with lipophilic, polyether, diarrheic shellfish toxins (DST) produced by marine microalgae. Originally, the DST complex comprised three groups of lipophilic toxins that often co-occur in natural samples of plankton and shellfish, and are detected all together by the conventional mouse bioassay (MBA): the okadaic acid (OA) and its analogues the dinophysistoxins (DTX), the yessotoxins (YTX), and the pectenotoxins (PTX) [2,3].

OA and its analogues, especially DTX-1 and DTX-2, are the most important DST toxins and cause inflammation of the intestinal tracts and diarrhea [4]. They are specific inhibitors of serine/threonine protein phosphatases 1 (PP1) and 2A (PP2A), two enzymes involved in the regulation of many cellular processes by modulation of protein phosphorylation/dephosphorylation degree [5,6]. In addition, OA and its analogues, including DTX-3, were also shown to have tumor promoting activity [7], and to exhibit several cellular effects both *in vitro* and *in vivo* (reviewed in [8]). Several toxicological studies showed that YTX exhibit lower potency for the inhibition of PP2A than OA and its analogues when administered orally [9,10]. Although YTX were also found to cause adverse pharmacological effects on cellular calcium regulation and phosphodiesterase coordination [11], they are no longer considered diarrheagenic and were removed from the original DST complex due to the fact that no related human intoxication has been reported to date [12]. Likewise, PTX, which are polyether-lactones, are no longer considered part of the DST complex [12], in spite of showing hepatotoxicity to mice following intraperitonial injection [4,13,14], and cytotoxicity in several mammalian cells [15] with antitumorigenic properties (reviewed in [16]). The PTX analogues, PTX secoacid (PTXSA) are not toxic to mice when administered orally [14,17,18]. YTX and its analogues are produced by the microalgae *Protoceritium reticulatum* [11], *Lingulodinium polyedrum* [19,20], and *Gonyaulax spinifera* [21]. OA and its derivatives are produced by some benthic species of the genus *Prorocentrum*, but mainly by species of the genus *Dinophysis* which also produce PTX [22].

The genus *Dinophysis* regroups over a 100 species of pigmented dinoflagellates, some of which have been shown to be mixotrophic [23,24]. Among these species of cosmopolitan, polymorphic, and mostly rare marine protists, typically exhibiting low cell densities of 10–10^2^ cells·L^−1^ and atypically occurring at 10^4^–10^5^ cells·L^−1^ in coastal waters [22,23,25], 12 have been found to produce OA, DTX, and/or PTX, and seven have been associated with DSP events (*D. acuminata*, *D. acuta*, *D. caudata*, *D. fortii*, *D. miles*, *D. ovum*, and *D. sacculus*) [26]. Species of *Dinophysis* form a small fraction of the microplankton community (1%–5%) and tend to aggregate in patchy thin layers, exceptionally forming red tides of more than 10^6^ cells·L^−1^ [26,27,28,29,30,31,32,33]. Nonetheless, DSP events associated with the toxins of *Dinophysis* spp. can emerge in any bivalve cultivation area covered by monitoring programs of both the cells and the toxins of *Dinophysis* [33].

The cellular toxic profile and content of *Dinophysis* spp. affect the magnitude of contamination of bivalves with LT. However, DSP events often occur in areas where several *Dinophysis* species with different toxin profiles are reported [26]. For instance, the contributions of blooms of *D. caudata*, co-occurring with or occurring after blooms of other toxigenic species such as *D. acuminata*, *D. sacculus* and *D. miles*, to the associated DSP events in Southern Europe, Northern Africa, Mexico, the Gulf of Mexico, South America, Southeast Asia, and Australia remain controversial [34,35,36,37,38,39,40,41,42,43,44,45,46,47,48]. Information on toxin profiles and toxin content of *D. caudata*, and other species of *Dinophysis*, is mainly available from cell concentrates and/or picked cells, due to difficulties to establish and maintain cultures of *Dinophysis* spp. [26,48]. Analyses of picked cells of *D. caudata*, from different locations, by HPLC-FLD and LC-MS showed the presence of OA and DTX-1 at moderate to high values [49], traces of OA and/or DTX-2 with high levels of PTX-2 [37], or only high levels of PTX-2 [50]. In addition, inter-annual variability in the toxin content of *D. caudata* picked from the same location was also found [37,50,51]. Red tides of *D. caudata* with associated fish mortality were reported in the Seto Inland Sea, Japan [52], and caused major DSP contamination in Singapore [53]. In a recent study, a monoclonal culture of *D. caudata*, isolated from western Japan, was found to be highly lethal, under controlled laboratory conditions, to Japanese scallops, *Patinopecten yessoensis*, and noble scallops, *Mimachlamys nobilis*, raising further questions regarding the toxicity of *Dinophysis* [54]. The recent successful cultivation of *D. caudata* [55] was crucial to understand the physiology and toxicology of this species, and other species that were also successfully cultured, namely *D. acuminata* [56], *D. fortii* [57], *D. infundibulus* [58], *D. tripos* [59,60], *D. acuta* [61], *D. cf. ovum* [62], and *D. sacculus* [63]. The production and accumulation of toxins in microalgal cells is controlled by several intrinsic and extrinsic factors [64,65], and temperature could be one of them, especially for *D. caudata* which is widely distributed in tropical and temperate neritic waters [33]. The present study considers the effect of seven experimental temperatures, covering the natural range of geographical distribution of *D. caudata*, on the growth and toxin production of a strain isolated from western Japan and maintained in a monoclonal culture.

## 2. Results and Discussion

### 2.1. Growth under Different Temperatures

**Figure 1:**
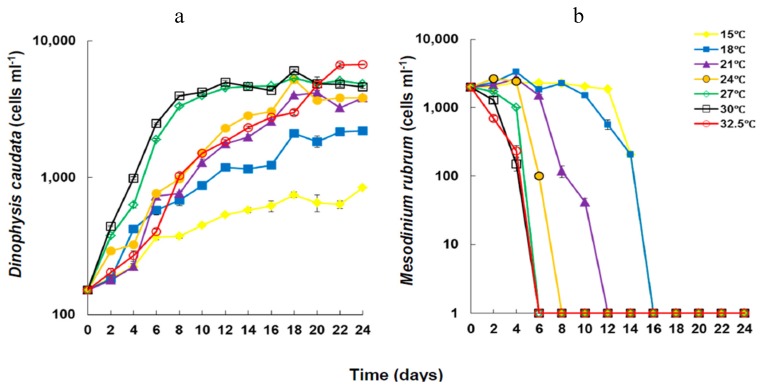
Changes in cell density of *Dinophysis caudata* (**a**), fed with *Mesodinium rubrum* (**b**), grown under different temperatures. Vertical bars denote the standard deviation (SD) of the mean (*n* = 3).

Cell densities of *Dinophysis caudata* increased significantly (Kruskal Wallis ANOVA, *p* < 0.05) under all experimental temperatures (Figure 1), reaching the highest final yield at 32.5 °C. Cell densities of *Mesodinium rubrum*, on the other hand, decreased rapidly with incubation time and increasing temperature, in a trend much similar to previous observations for similar temperatures [55], *i.e.*, its exponential decline occurred more rapidly at temperatures >27 °C. Maximal yield in *D. caudata* cultures was significantly higher at temperatures above 21 °C (Table 1).

**Table 1:** Results of the multiple comparisons test *H* (Kruskal Wallis Anova) for the effects of temperature (°C) on cell density of *Dinophysis caudate*.

	Temperature (°C)						
	15	18	21	24	27	30	32.5
15							
18	NS						
21	*	NS					
24	***	NS	NS				
27	***	***	*	NS			
30	***	***	*	NS	NS		
32.5	***	NS	NS	NS	NS	NS	

NS: Non-significant, * *p* < 0.05; *** *p* < 0.001.

**Figure 2:**
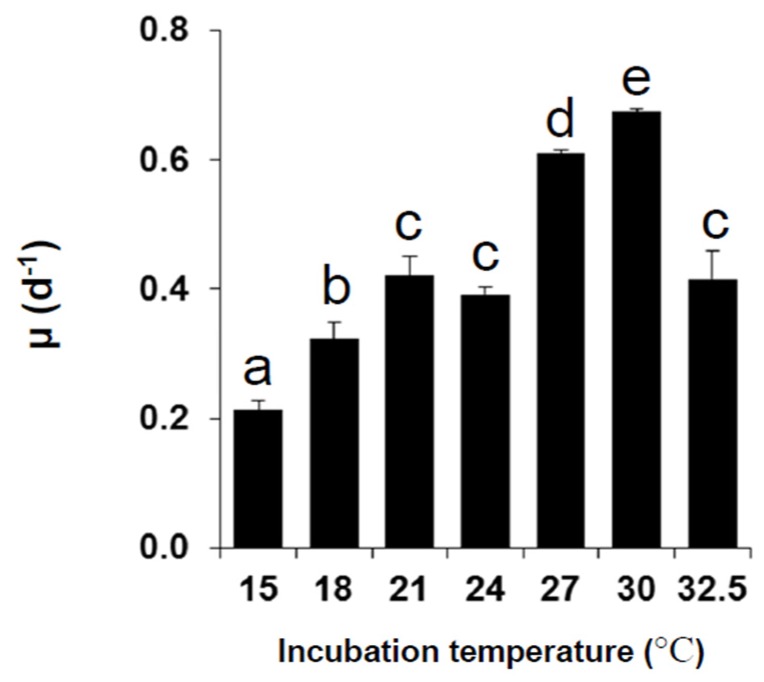
Average specific growth rates of *Dinophysis caudata* during the exponential growth phase under different experimental temperatures. Vertical bars denote the standard deviation (SD) of the mean (*n* = 3). Different letters indicate significant differences among treatments (ANOVA, Neuwman-Keuls, *p* < 0.05).

During exponential growth phase (EG), the specific growth rate (µ) of *D. caudata* showed significant difference in response to different temperatures (Figure 2). It ranged from 0.21 ± 0.01 day^−1^ at 15 °C to 0.67 ± 0.00 day^−1^ at 30 °C; increasing significantly from 15 °C to 30 °C, and then decreasing at 32.5 °C. The specific growth rate of *D. caudata* was within the range of specific growth rates reported in previous studies for the same species isolated from Japan, and other *Dinophysis* species (*D. acuminata*, *D. acuta*, *D. fortii*, *D. infundibulus*, *D. norvegica*, *D. tripos*, and *D. sacculus*), from both *in situ* and culture estimates (reviewed in [66]). It was optimal under 24, 27, and 30 °C, which reflects the natural distribution of *D. caudata* in tropical and warm sub-tropical areas, with higher cell density, and exceptional blooms, occurring in warmer tropical areas [33]. Temperatures lower than 21 °C and above 30 °C seem to be outside the lower and higher boundaries of optimal growth of *D. caudata*, although insufficient acclimation at the lowest and the highest temperatures should not be excluded. In a previous study, increasing temperature was shown to enhance maximal yield and specific growth of *D. acuminata* in culture experiments, also reflecting its cosmopolitan geographical distribution [67,68].

Temperature affects the physiology, notably the metabolic activity of phytoplankton, which results in increased food uptake and active cell division [69]. Temperature does also affect the feeding behavior of *Dinophysis* spp., and the swimming behavior and speed of both prey and predator resulting in higher growth rates with increasing temperature [65,70]. The densities of the prey *M. rubrum* decreased rapidly at temperatures >21 °C, especially at temperatures exceeding 27 °C under which the rapid decrease occurred within less than six days, coincident with EG of *D. caudata*. The difference in growth rates during EG should have been linked to active grazing sustaining the mixotrophic growth of *Dinophysis* through both photosynthesis, and essential nutrients and growth factors obtained via prey consumption [71,72,73,74,75,76,77,78,79]. The swimming speed of the prey *M. rubrum* decreases with decreasing temperature [75]. Therefore, the lower growth rates at lower temperatures in spite of the longer availability of the prey should have been related to the direct influence of temperature on the physiology of *D. caudata* and thus its growth. It should also be noted that growth rates are influenced by both extrinsic and intrinsic factors, including the experimental conditions but also genetic variability inherent to the strain of *Dinophysis* and to the prey itself [55].

### 2.2. Toxin Content and Production under Different Temperatures

**Figure 3:**
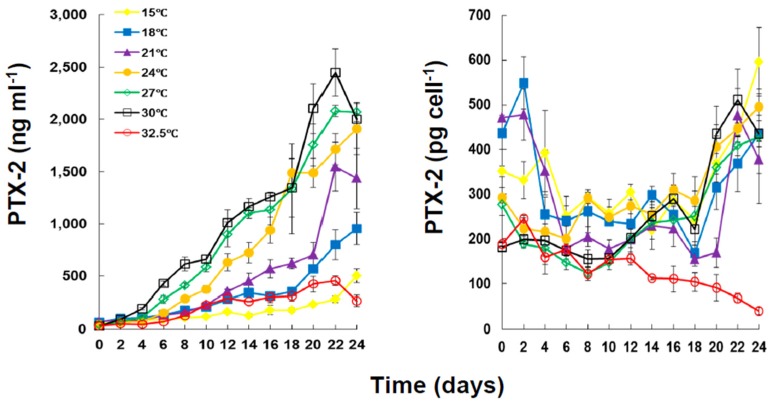
Concentration of PTX-2 (ng·mL^−1^) and cellular production (particulate plus dissolved) of PTX-2 (pg·cell^−1^) in cultures of *Dinophysis caudata* grown at different temperatures. Vertical bars denote the standard deviation (SD) of the mean (*n* = 3). PTX = pectenotoxins.

The strain of *D. caudata* used in this study produced only PTX-2 (Figure 3). The concentration of PTX-2 in the culture was affected by both incubation period (Mann-Whitney U test, *p* < 0.05) and temperature (Kruskal Wallis ANOVA, *p* < 0.05).

There was a significant increase in the toxin content of the culture with increased incubation time (Mann-Whiney U Test, *p* < 0.05) associated with increased cell density of *D. caudata*. Within 22–24 days of incubation, the concentration of PTX-2 reached the highest value of 2.45 ± 0.25 × 10^3^ ng·mL^−1^ at 30 °C. The lowest concentration of PTX-2 (0.45 ± 0.05 × 10^3^ ng·mL^−1^) throughout the experimental period was registered for incubation at 32.5 °C, a temperature under which PTX-2 concentration decreased. The concentration of PTX-2 in the culture was significantly different under temperatures ≤18 °C and ≥21 °C, but not significantly different under 21, 24, 27, and 30 °C. On the other hand, the concentration of PTX-2 in the culture was significantly different under 32.5 °C, and 24, 27 and 30 °C (Table 2). The PTX-2 cellular toxin production of *D. caudata*, which corresponds to the total toxin production (particulate plus dissolved) per cell per mL of culture, showed variation with growth phase and temperature (Figure 3). Except for the decreased cellular toxin production for incubation at 32.5 °C, from 188.45 ± 5.16 pg·cell^−1^ to 39.84 ± 7.84 pg·cell^−1^, there was a decrease at the beginning of the incubation period until day 6, corresponding to early to mid-exponential growth, a stabilization between 6 and 18 days, corresponding to late-exponential to mid-stationary growth, and then an increase between 18 and 24 days, corresponding to late stationary growth, to levels higher than the ones registered during the first six days.

**Table 2:** Results of the multiple comparisons test *H* (Kruskal Wallis ANOVA) for the effects of temperature (°C) on PTX-2 concentration of *Dinophysis caudate*.

	Temperature (°C)						
	15	18	21	24	27	30	32.5
15							
18	NS						
21	*	NS					
24	***	NS	NS				
27	***	NS	NS	NS			
30	***	*	NS	NS	NS		
32.5	NS	NS	NS	*	*	*	

NS: Non-significant, * *p* < 0.05; *** *p* < 0.001. PTX = pectenotoxins.

**Figure 4:**
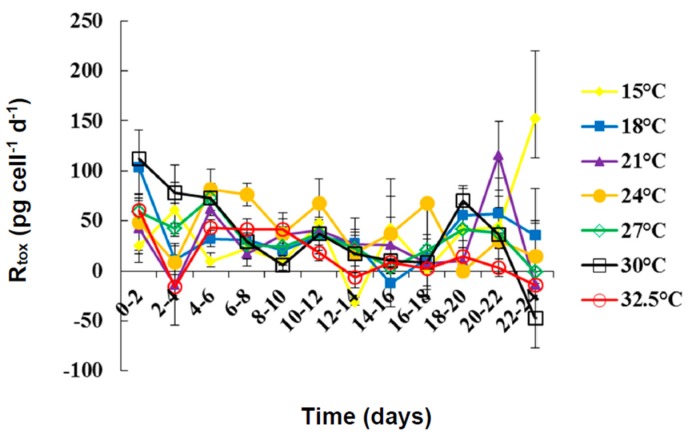
Net toxin production (R_tox_) of PTX2 by *Dinophysis caudata* grown at different temperatures. Vertical bars denote the standard deviation (SD) of the mean (*n* = 3).

The average net toxin production (R_tox_) of *D. caudata* (Figure 4) ranged from 35.16 ± 12.87 pg·cell^−1^·day^−1^ at 15 °C to 16.29 ± 7.24 pg·cell^−1^·day^−1^ at 32.5 °C (Table 3). The R_tox_ was significantly affected by the incubation period (Kruskal Wallis ANOVA, *p* < 0.001) but not by temperature. Except for the peak at 15 °C from day 16–24, the R_tox_ decreased with incubation period. The increase in the concentration of PTX-2 in the culture medium with incubation period and the variation of R_tox_ of PTX-2 with growth phase and temperature were, therefore, most probably related to differential release of PTX-2 into the culture medium than to an actual differential cellular production of PTX-2 with growth phase and temperature. The cellular toxin content (CTC) of PTX-2 and DTX-1 of *D. acuminata* in culture were also found to be unaffected by experimental temperature whereas the CTC of OA increased significantly with increasing temperature [67].

**Table 3:** Net toxin production (R_tox_, pg·cell^−1^·day^−1^) of *Dinophysis caudata* during the entire experimental growth under different temperatures.

		Temperature (°C)						
		15	18	21	24	27	30	32.5
R_tox_	Min	−31.67	−12.31	−14.77	0	−1.35	−47.60	−16.04
	Max	152.04	103.32	115.33	81.39	72.86	112.30	60.65
	Mean	35.16	33.98	29.46	22.93	32.06	35.79	16.29
	SE	12.87	8.35	101.63	8.01	6.10	12.27	7.24

In another report, the intracellular amount of PTX-2 of *D. acuminata* and *D. fortii* were found to be equal to the total amount of PTX-2 in culture experiments, whereas the intracellular amount of OA and DTX-1 were different [66]. It seems that the production, retention/release of toxin, at least PTX-2, varies among species of *Dinophysis* and/or strains of the same species.

**Figure 5:**
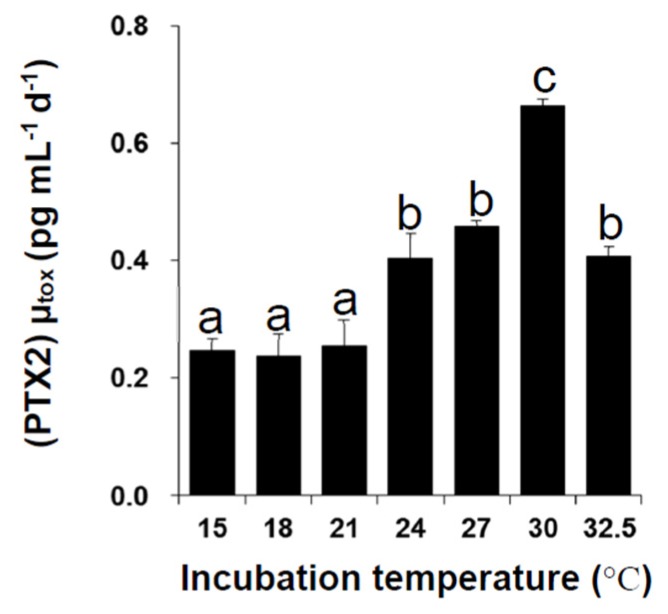
Average specific toxin production rate (µ_tox_) of PTX-2 by *Dinophysis caudata* during the exponential growth phase, cultured under different temperatures. Vertical bars denote the standard deviation (SD) of the mean (*n* = 3).

Early HPLC-FLD analyses, that did not search for PTX, showed very low CTC of OA (0.07–0.14 pg·cell^−1^) in *D. caudata* from Singapore [53,80], and moderate to high CTC of OA (7.9–56.5 pg·cell^−1^) and DTX1 (7.2–53.9 pg·cell^−1^) from the Philippines [49]. Recent LC-MS analyses, however, showed that PTX-2 is the dominant or the only toxin present in Northwest Spain with CTC ranging from 50 to 120 pg·cell^−1^ accompanied by traces of OA and/or DTX-2 [42,50]. Differential variations of CTC of OA, DTX-1, and PTX-2 with growth phase were reported for cultured *D. acuminata* from northeastern USA, with a significant decrease of PTX-2 as the culture aged [68]. On the other hand, the average specific toxin production rate (µ_tox_) during exponential growth phase showed significant difference among temperature (Figure 5). While µ_tox_ was not significantly different at the lower temperatures, 15 to 21 °C, it increased significantly from 24 °C, reaching a peak of 0.66 ± 0.01 day^−1^ at 30 °C, and then decreased at 32.5 °C.

**Figure 6:**
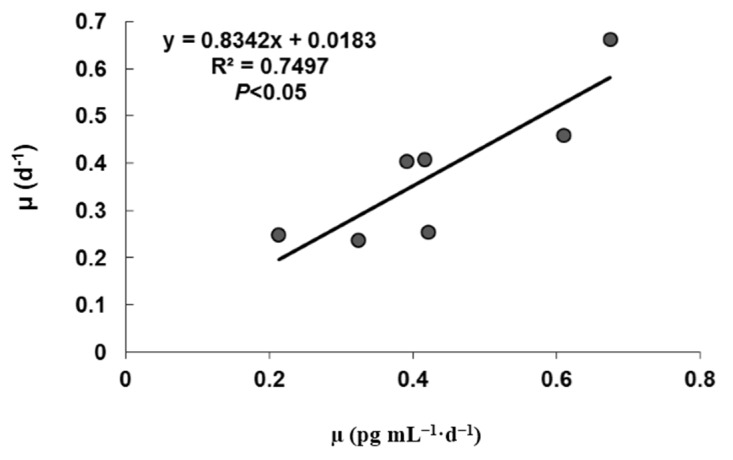
Relationship between specific toxin production rate (µ_tox_) and specific growth rate (µ) of *Dinophysis caudata* during the exponential growth phase. Averaged values were plotted.

These results suggest that, during early exponential growth, temperature is a direct contributor to the production of PTX-2, but that PTX-2 production is also related to growth. Indeed, µ_tox_ was significantly correlated to µ in cultures of *D. caudata* during EG (Figure 6) but also over the entire incubation period, all temperatures considered (*r* = 0.50, *p* <0.001). But this correlation was most significantly contributed from the µ_tox_ at 27 °C (*r* = 0.80, *p* <0.01) and 30 °C (*r* = 0.82, *p* <0.01). In a previous study, temperature was found to affect PTX-2 production of a strain of *D. acuminata* from Japan [65], whereas another report showed that both temperature and light did not affect the production of PTX-2 of another strain of *D. acuminata* form USA [68]. Several factors may interactively influence the production of PTX-2 in *D. caudata*, including the prey organism, growth, and temperature. In the light of these results, further studies should be considered to understand the factors that influence toxin production in cultures of *D. caudata*.

## 3. Experimental Section

### 3.1. Isolation and Maintenance of Clonal Strains

The cryptophyte *Teleaulax amphioxeia* and the marine ciliate *Mesodinium rubrum* were isolated from Inokushi Bay, Oita Prefecture, Japan (32°47′ N, 131°53′ E), in 2007 [55,57]. Cultures of *M. rubrum* were maintained at 18 °C, by weekly re-inoculations of 50 mL of the culture into a 100 mL of modified f/2 medium [81,82,83], with the addition of 50 µL of *T. amphioxeia* culture as prey, under an irradiance of 100–150 µmol photons m^−2^·s^−1^ provided from cool white fluorescent lamps with a 12 h light: 12 h dark cycle. The culture medium was prepared with 1/3 nitrate, phosphate, and metals and 1/10 vitamins based on the enrichment of natural seawater collected from Hiroshima Bay (salinity adjusted to 30). Cultures of *T. amphioxeia* were maintained under the same conditions as those of *M. rubrum*, by re-inoculating 0.3 mL of the culture into 150 mL of the modified f/2 medium. Cultures of *Dinophysis caudata* were established from single cells of *D. caudata* isolated from a seawater sample collected from Nagasaki Prefecture. The cells did not have 2 tailing flagella (planozygotes), hence the established cultures, from a 1n-cell, were monoclonal. Cultures of *D. caudata* were initiated by incubating individual cells in a 12-well microplate in the culture medium with *M. rubrum*. Cultures were maintained by incubation in 10 mL culture tubes under each experimental temperature, for 20 days. The volume of the cultures was gradually increased to 150 mL through monthly re-inoculations into *M. rubrum* cultures at 21–23 °C in a 250 mL carbonate Erlenmeyer flask [55].

### 3.2. Growth Experiments

The effects of temperature on toxin production were investigated at seven temperatures (15, 18, 21, 24, 27, 30, and 32.5 °C), under the same conditions specified for culture maintenance, except for temperature. Both *M. rubrum* and *D. caudata* cultures were pre-conditioned to each experimental temperature prior to growth and toxin measurements for 20 days. The *M. rubrum* cultures grown at each temperature were collected at the late exponential growth phase (*ca.* 8.5 × 10^3^ cells·mL^−1^), and then diluted with the culture medium to an initial concentration of *ca.* 2 × 10^3^ cells·mL^−1^. Cultures of *D. caudata* for each temperature were added to the matching culture of *M. rubrum* for a final concentration of 150 cell·mL^−1^ of *D. caudata*. Every 2 days, the cultures were shaken and samples collected for cell counts (0.5 mL, in triplicate), and DSP and lipophilic toxin analysis (1.0 mL, in triplicate). Cells of *M. rubrum* and *D. caudata* were counted using an inverted microscope. Samples for toxin analysis were kept at −20 °C.

The specific growth rate (µ, day^−1^) of *D. caudata* was calculated from the exponential growth phase (EG), between sampling intervals (*t*, 2 days), as follows [84]:

(1) $$µ=\;\frac{\text{ln}\left(N_{2}/N_{1}\right)}{t}$$

where, *N*_1_ and *N*_2_ are the cell densities of *D. caudata* at time 1 and time 2, respectively. *t* is the experimental time (days), and µ is the specific growth rate (day^−1^). The mean specific growth rates of *D. caudata* were determined from the slope of the linear regression of the natural logarithm of cell density *versus* incubation time during the exponential growth phase.

### 3.3. Toxin Analyses

The solid phase extraction (SPE) procedure was used for toxin analysis [65,85]. Following a 2 min sonication, each sample was loaded on a Sep-Pak C18 cartridge column (Waters, Milford, MA, USA), previously preconditioned with methanol (5 mL) and distilled water (10 mL). Sea salt was then removed by washing the cartridge with 5 mL distilled water, and the toxins were eluted with 5 mL methanol. Following evaporation, the residue was dissolved in 200 µL methanol, and an aliquot of the solution was directly analyzed by liquid chromatography-tandem mass spectrometry (LC-MS/MS) analysis.

The LC-MS/MS analysis was carried out according to a previous method with slight modifications [86]. The lowest detection limits for OA, DTX1, and PTX2 were 0.6, 0.6, and 1.6 ng·mL^−1^, respectively. These levels are equivalent to 1.2 pg·cell^−1^ of OA/DTX1 and 3.2 pg·cell^−1^ of PTX2, when 100 cells of the toxic plankton were analyzed using our LC-MS/MS method.

The specific toxin production rate (µ_tox_, pg·mL^−1^·day^−1^) was calculated based on Equation (1), by substituting *N*, the cell density, by *T*, the toxin concentration, between two consecutive sampling points, during EG phase, as follows:

(2) $${µ}_{\text{tox}}=\;\frac{\text{ln}\left(N_{2}T_{2}/N_{1}T_{1}\right)}{t}$$

To account for cell growth rates on toxin production, the net toxin production rate (R_tox_; pg·cell^−1^·day^−1^) was determined from the following equation [81,87]:

(3) $$R_{\text{tox}}=\;\frac{\left(T_{2}-T_{1}\right)}{N_{m}\;\times \;t},\;N_{m}=\;\frac{\left(N_{2}-N_{1}\right)}{\text{ln}\left(N_{2}/N_{1}\right)}$$

where, *T*_1_ and *T*_2_ are the toxin concentrations and *N*_1_ and *N*_2_ are the cell densities of *D. caudata* both at the first and subsequent sampling time, respectively, and *N_m_* is the geometric mean density of *D. caudata* during the sampling period.

### 3.4. Data Analysis

Normality (Shapiro-Wilk) and homoscedasticity (Cochran test and Bartlett test) were checked *a priori*. When the assumptions of the normal distribution were met, multivariate or factorial ANOVA were used to test the effects of incubation period and temperature followed by the *post-hoc* test, Fisher’s Least Significant Difference (LSD). Otherwise, the non-parametric Kruskal-Wallis ANOVA was considered, followed by the multiple comparisons H test to assess the level at which the significant effects occurred. The effects of incubation period and temperature on toxin concentration and net toxin production were tested with Spearman rank order correlations followed by the Mann-Whitney U test. Differences between replications, for all data sets, were not significant. Three levels of significance were considered, α = 0.05, 0.01, and 0.001.

## 4. Conclusions

In conclusion, cell densities of *D. caudata* increased significantly with increasing temperature, with the highest yields observed under 27, 30, and 32.5 °C. It is interesting that *D. caudata* only produced PTX-2. Because PTX-2 is rapidly converted to non-toxic PTX-2 seco acid in many bivalve species, except for Japanese scallops *Patinopecten yessoensis* [85,86,88], appearance of *D. caudata* even at high densities could not be problematic for many bivalve species in terms of shellfish toxin regulation by both mouth bioassay and LC-MS/MS. On the other hand, PTX-2 still has attracted attention for toxicological and pharmacological reasons. Therefore, results obtained in the present study would be very useful for optimization of mass production of PTX-2 through large scale cultures of *D. caudata*.
